# TRANSITION IN FAMILY RELATIONS IN IMMIGRANT FAMILIES: THE CASE OF CHINESE OLDER IMMIGRANTS IN THE UNITED STATES

**DOI:** 10.1093/geroni/igz038.117

**Published:** 2019-11-08

**Authors:** Man Guo, Meredith stensland, Mengting Li, XinQi Dong

**Affiliations:** 1 University of Iowa, Iowa, United States; 2 university of Texas at Austin, Austin, Texas, United States; 3 Rutgers, The State University of New Jersey, New Brunswick, New Jersey, United States

## Abstract

Using panel data of 2,604 Chinese older immigrants in Chicago over a two-year period, this study examined continuity and changes in intergenerational relationship patterns and their mental health implications. Latent transition analysis revealed five types of family relations: traditional, modified traditional, coresiding-unobligated, independent, and detached. Over the two years, about 43% of the respondents shifted to a different relationship type, with the most common changes being shifting into modified traditional or independent relations, or from detached relations. Controlling for baseline socio-demographic, acculturation, mental health variables, and variables representing life transitions over time, having detached relations was related to greater depressive symptoms in two years and having modified traditional relations was associated with better quality of life at the follow up. The findings revealed heterogeneity and fluidity of intergenerational relations among older immigrant populations and point to the important role of family relations in their well-being.

